# 546 Winning the Race Against Pathogens: A Regional Burn Center Experience with a Cleansing Protocol

**DOI:** 10.1093/jbcr/irad045.143

**Published:** 2023-05-15

**Authors:** David Dries, Sam Miotke, Emily Popma, Mark Johnston, Bradley Rogers, Mary Anne Obst

**Affiliations:** Regions Hospital, St Paul, Minnesota; Regions Hospital, St Paul, Minnesota; Regions Hospital, St Paul, Minnesota; Regions Hospital, St Paul, Minnesota; Regions, St Paul, Minnesota; Regions Hospital, St Paul, Minnesota; Regions Hospital, St Paul, Minnesota; Regions Hospital, St Paul, Minnesota; Regions Hospital, St Paul, Minnesota; Regions Hospital, St Paul, Minnesota; Regions, St Paul, Minnesota; Regions Hospital, St Paul, Minnesota; Regions Hospital, St Paul, Minnesota; Regions Hospital, St Paul, Minnesota; Regions Hospital, St Paul, Minnesota; Regions Hospital, St Paul, Minnesota; Regions, St Paul, Minnesota; Regions Hospital, St Paul, Minnesota; Regions Hospital, St Paul, Minnesota; Regions Hospital, St Paul, Minnesota; Regions Hospital, St Paul, Minnesota; Regions Hospital, St Paul, Minnesota; Regions, St Paul, Minnesota; Regions Hospital, St Paul, Minnesota; Regions Hospital, St Paul, Minnesota; Regions Hospital, St Paul, Minnesota; Regions Hospital, St Paul, Minnesota; Regions Hospital, St Paul, Minnesota; Regions, St Paul, Minnesota; Regions Hospital, St Paul, Minnesota; Regions Hospital, St Paul, Minnesota; Regions Hospital, St Paul, Minnesota; Regions Hospital, St Paul, Minnesota; Regions Hospital, St Paul, Minnesota; Regions, St Paul, Minnesota; Regions Hospital, St Paul, Minnesota

## Abstract

**Introduction:**

The skin is the first immune defense mechanism, and it functions as a barrier against various microorganisms. Burn injury will compromise this important barrier. Infections are the primary factor contributing to burn mortality, resulting in >50% of deaths in burn patients. In 2020, our Adult and Pediatric Burn Center instituted a pure Hypochlorous Acid (pHA) antimicrobial protocol to enhance skin barrier function.

**Methods:**

We conducted a retrospective analysis on the effect of the introduction of the pHA protocol on the number of reportable infections in our Burn Center. The period of observation in this report is 2017-2021. Beginning in 2020, adults and children received topical wound care and bathing with our pHA protocol. No dilution of the product was used. The product was placed into a sterile basin and the patient was cleaned head to toe, including the genitalia and the wounds. Since invasive procedures such as placement of central venous catheters or urinary collection catheters may be a conduit of infection, the present report includes data regarding the number of patients experiencing clostridium difficile, central line-associated blood stream infection and catheter-associated urinary tract infections.

**Results:**

From 2017-2021, our Burn Center admitted 2,223 patients. We computed a percent change in infections from before (2017-2019) to after (2021) the pHA protocol was implemented. We used 2021 because this was the first full year after the pHA protocol was introduced. We subtracted the total number of infections in 2021 from the average number of infections per year in 2017-2019 and divided by the average number of infections per year in 2017-2019. We found a 55% decrease in infections after the pHA protocol was introduced. There was no evidence of toxicity in adult or pediatric patients associated with care during introduction of the pHA protocol, including skin graft sites.

**Conclusions:**

This analysis indicates a notable decrease in infections. The pHA procedure shows no evidence of viral, fungal or bacterial pathogens causing significant issues with the studied patient group. This analysis does not determine whether the decrease in infections is clinically or statistically significant.

**Applicability of Research to Practice:**

Reducing infections in burn patients which can be life saving.

